# Potential Mechanism of Long-Term Immobilization of Pb/Cd by Layered Double Hydroxide Doped Chicken-Manure Biochar

**DOI:** 10.3390/ijerph20010867

**Published:** 2023-01-03

**Authors:** Xiaoxian Zhang, Tingran Liu, Jichen Zhang, Ling Zhu

**Affiliations:** State Key Laboratory of Pollution Control and Resources Reuse, Department of Environmental Science, Shanghai Institute of Pollution Control and Ecological Security, College of Environmental Science and Engineering, Tongji University, Shanghai 200092, China

**Keywords:** chicken-manure biochar, layered double hydroxide, heavy metals, smelting site

## Abstract

Layered double hydroxide (LDH)-doped chicken-manure biochar (CMB) with long-term stability was synthesized to immobilize Pb/Cd. MgAl-Cl-LDH-doped CMB (MHs) showed prominent long-term oxidation resistance and the least biodegradation sensitivity. Efficient Pb/Cd adsorption was observed on MHs, and the maximum adsorption capacities of Pb(II)/Cd(II) reached 1.95 mmol/g and 0.65 mmol/g, respectively. Precipitation and isomorphous substitution were identified as the key adsorption mechanisms, which formed highly stable Pb/Cd species (PbAl-CO_3_-LDH, Pb_3_(OH)_2_CO_3_, CdAl-Cl-LDH and CdCO_3_). Pb(II) and Cd(II) precipitated with CO_3_^2−^ in MHs; meanwhile, Mg(II) and Ca(II) in LDH layers were substituted by Pb(II) and Cd(II) respectively. Therefore, MHs had the potential for long-term stability of Pb/Cd. Moreover, complexation and electrostatic adsorption also contributed to the Pb/Cd immobilization to a certain extent. When 5% MHs (*w*/*w*) was applied to Pb/Cd contaminated smelting site soils, the soil pH increased from 5.9 to 7.3. After applying MHs for 25 d, the content of bioavailable Pb(II) and Cd(II) decreased by 98.8% and 85.2%, respectively, and the content of soluble Pb and Cd dropped by 99.5% and 96.7%. This study paves the way for designing a novel LDH doped CMB as efficient Pb/Cd immobilizers for smelting site soils.

## 1. Introduction

China is one of the biggest producers and consumers of non-ferrous metals in the world, with a production capacity in 2021 above 61.8 million tons [1]. Large-scale smelting activities make the soil of the smelting site suffer from the continuous input of high levels of harmful heavy metals [2,3,4,5,6], which pose a threat to the soil ecosystem and human health. According to recent reports [7,8], Pb(II) and Cd(II) are the most widespread heavy metals in smelting site soils in China. Pb(II) is non-biodegradable and highly carcinogenic [9,10,11], while Cd(II) is highly mobile and toxic [12,13]. Thus, there is an urgent need for timely immobilization of Pb(II) and Cd(II) in smelting site soils using cost-effective agents.

Biochar, a highly aromatic solid with porous structure and large specific surface area, is favorable for Pb/Cd immobilization. The carbonate, O-containing functional groups and silicates are the primary components responsible for ameliorating soil acidification and reduction of bioavailable cationic heavy metals through electrostatic attraction, precipitation, complexation and ion exchange [14,15]. Furthermore, biochar may also enhance the water holding capacity of soil and facilitate nutrient absorption of plants in soils [16]. In this regard, the conversion of chicken manure, a type of common biowaste, into biochar for heavy metal immobilization could be a win-win strategy. Chicken-manure biochar (CMB), sufficient in oxygen and nitrogen, and containing functional groups, may complex with Pb/Cd [17,18]. However, compared with plant or animal-derived biochar, CMB is often blamed for the relatively smaller specific surface area and lower content of Ca-containing minerals [19,20], which may to some extent affect the immobilization efficiency of heavy metals. In addition, the long-term effectiveness of CMB as heavy metal immobilizers should be considered. Although biochar has a far longer residence time than its precursor biomass, the regulation of biochar on soil acidity would almost disappear after 10 years of application. In the long run, the content of aluminum or calcium-containing minerals that improve the antioxidant capacity of biochar might also decrease [21,22]. Studies on the enhancement of CMB immobilizing ability have thus drawn increasing attention.

Layered double hydroxides (LDHs), i.e., hydrotalcite-like lamellar minerals, are composed mainly of light metals, such as Mg, Al, and/or Ca, and hydroxyl groups, best known for their strong pH-buffer ability [23]. LDH-doped CMB (MHs) is likely to assist CMB to function better in the following two ways. First, light metals contained in LDH may improve the long-term stability of CMB. Secondly, in the past four years, attempts to introduce LDHs into biochar structure to achieve better Pb/Cd adsorption performance have sprung up because MgAl-LDH is able to both precipitate with Pb(II) by forming Pb(OH)_2_, PbCO_3_PbCl_2_, Pb(OH)Cl, and Pb_3_(OH)_2_CO_3_, and isomorphically substitute Pb(II), while CaAl-LDH targets Cd(II) for isomorphic substitution [24,25,26,27,28]. It has therefore been hypothesized that biochar doped by MgAl-LDH and CaAl-LDH may immobilize Pb/Cd as a super stable agent. In order to enhance adsorption performance of LDH-doped biochar, many researchers have tried to find out a more suitable type of LDH to equip the biochar matrix [29,30], but very few have focused on optimizing the ratio of divalent metals and trivalent metals in LDH layers [31], and even fewer have noticed that the LDH/biochar ratio would matter [32]. The remaining research gaps need to be bridged.

Hence, the objective of this study is to investigate how to optimize the preparation of a novel stable Pb/Cd immobilizer, i.e., LDH-doped CMB, containing both MgAl-LDH and CaAl-LDH, by a simple co-precipitation method with varying ratios of LDH/CMB. Short-term stability and long-term stability were examined carefully on CMB and LDH-doped CMB. X-ray diffraction (XRD), Scanning Electron Microscope with Energy Dispersive Spectroscopy (SEM/EDS), Zeta potential, Fourier Transform Infrared Spectroscopy (FTIR), X-ray photoelectron spectra (XPS) and micro-X-ray fluorescence (XRF) data were used to explore the complicated immobilization mechanism. A typical heavy metal smelting site in Gansu, China, was selected for evaluation of Pb/Cd co-immobilization effect of synthetic materials.

## 2. Materials and Methods

### 2.1. Material Preparation and Characterization

The CMB used in this study was produced from the pyrolysis of air-dried chicken manure at 500 °C in an oxygen-limited environment for 4 h (Zhejiang Eco Environmental Tech Co., Ltd., Zhejiang, China). The CMB was washed with deionized water until the pH value was constant. LDH-doped CMB was synthesized by the co-precipitation method [32,33] with 0.15 mol MgCl_2_, 0.075 mol AlCl_3_ and 5 g, 15 g or 30 g CMB (see details in Text S1 and Figure S1), and the obtained MgAl-Cl-LDH-doped CMB was denoted as MH1, MH2, MH3, respectively. MgAl-Cl-LDH was also prepared by co-precipitation. All samples (CMB, MH1, MH2, MH3 and MgAl-Cl-LDH) were ground and sieved (0.45 mm) for further study.

The analysis of total C, H, N were conducted with an elemental analyzer (Vario EL III, Elementar, Hanau, Germany), and N content was used for quantifying the load of CMB in MH1, MH2 and MH3 (CMB%). Elements such as Mg, Ca, Al, K, Fe, Na and P were determined by inductively-coupled plasma optical-emission spectrometer (ICP-OES, Agilent 720ES, Palo Alto, CA, USA) after digestion by 17 M HNO_3_/8.8 M H_2_O_2_. The XRD (Bruker D8 Advance, Borken, Germany) pattern of each material was performed with CuKa-radiation, within the 2θ scanning range of 10–80°. The micropore specific surface area (microSSA) and pore volume of CMB and MH2 were tested with CO_2_ at 195 K after being outgassed under vacuum at 393 K for 6 h by 3Flex 5.0.2 micropore analyzer (Micromeritics, Norcross, GA, USA). The surface morphology was gained by the SEM (Carl ZEISS Sigma 300, Jena, Germany) equipped with Xplore EDS detector (Oxford Instruments, Oxfordshire, UK). Zeta potential was observed on Malvern Zetasizer Nano ZS90 instrument (Malvern, UK) to obtain pH_zpc_ (the pH at the zero point of charge). FTIR were collected in the range of 400–4000 cm^−1^ by Nicolet 5700 (Thermo Scientific, Waltham, CA, USA). Thermogravimetric/derivative thermogravimetric (TG/DTG) test was performed by PerkinElmer STA 8000 thermal analyzer (Waltham, MA, USA) at 30–800 °C in N_2_ (flow rate = 10 °C/min). An XRF examination was conducted in Beijing Synchrotron Radiation Facility (BSRF, 4W1B Beamline). The grounded samples of MH2 particles on Kapton tape were scanned 1000 μm stepwise with a spot-size ~50 μm in diameter. Spearman correlation was then performed in order to consider non-linear responses between different elements detected by XRF.

### 2.2. Measurement of Material Stability

The stability of biochar-containing materials normally refers to their ability to resist decomposition. Stability in water was assessed by quantitative variance of particle size distribution and zeta potential of fresh materials and materials dispersed in water (1.0 g/L) for 2 d. D_50_ (the value of the particle diameter at 50% in the cumulative distribution) was used for particle size quantification at pH 7.

Long-term stability in soils relied on a TG analysis at 30–1000 °C in O_2_ (flow rate = 10 °C/min). R_50, material_ was chosen to evaluate the oxidation recalcitrance of C-containing materials based on TG results as stated by Harvey et al. [34].

R_50, material_ = T_50, material_/T_50, graphite_ × 100% (1)

where T_50, material_ and T_50, graphite_ are the temperature values corresponding to 50% weight loss during TG test under O_2_ atmosphere, respectively. T_50, graphite_ was 844 °C according to Yang et al. [35].

### 2.3. Batch Adsorption Experiments of Pb/Cd

#### 2.3.1. Adsorption Isotherms

The adsorption experiment was carried out in 30 mL flasks with 10 mg adsorbents and 20 mL solution with varied initial concentrations of Pb(II) or Cd(II), prepared by Pb(NO_3_)_2_ (AR, ≥99.0%) or Cd(NO_3_)_2_·4H_2_O. The background ionic strength was 10 mM NaNO_3_. Initial pH in solutions was adjusted to 5.0, and pH values at equilibrium were recorded. After being shaken in a mechanical shaker at 180 rpm for 36 h (25 °C), the flasks were withdrawn and the inside mixtures were filtered by 0.22 μm pore size PTFE membrane filters. The concentration of Ca, Mg and Pb or Cd in solution was determined by ICP-OES. The adsorption capacity of Pb/Cd was calculated by the following equation:

Q_e_ = (C_0_ − C_e_)·V/m, (2)

where Q_e_ is the adsorption capacity (mmol/g), C_0_ is the initial concentration (mmol/L), C_e_ denotes the equilibrium concentration (mmol/L), V is the volume of solution (L) and m is the mass of adsorbent used (g).

Langmuir (Equation (3)) and Freundlich (Equation (4)) models were used to simulate adsorption isotherms on CMB, MH1, MH2 and MH3.

Q_e_ = Q_m_·K_L_·C_e_/(1 + K_L_·C_e_), (3)

Q_e_ = K_F_·C_e_^1/n^, (4)

where Q_e_ is the adsorption capacity (mmol/g), Q_m_ is Langmuir maximum capacity (mmol/g), K_L_ is Langmuir equilibrium constant (L/g), C_e_ is the equilibrium concentration (mmol/L), n is Freundlich linearity constant and K_F_ is Freundlich affinity coefficient (g^(1−n)^/L^−n^/g). The relationship of CMB% and Q_m_ was also explored (Text S2).

For isothermal experiments for a binary metal system, 10 mg MH2 was added into 20 mL solution with 0–2.4 mmol/L iso-stoichiometric Pb(II)/Cd(II) at pH of 5.0. The background ionic strength was adjusted by 10 mM NaNO_3_.

#### 2.3.2. The Effect of pH on Adsorption

In order to identify how pH value affects Pb/Cd adsorption capacity and mechanisms on LDH-doped CMB, adsorption of Pb (initial pH ranging for 2.0 to 6.0) or Cd (initial pH ranging for 2.0 to 8.0) onto MH2 was conducted, and initial pH was adjusted by HNO_3_ or NaOH prior to the addition of MH2. The final pH value was measured by using a pH meter (PHS-3C, INESA, Shanghai, China).

#### 2.3.3. Post-Sorption Characterization

Adsorption products were characterized in order to explore the mechanism behind. For reference, Pb adsorbed on CMB (CMB + Pb) and Cd adsorbed on CMB (CMB + Cd) were analyzed by FTIR. Grounded particles of Pb adsorbed on MH2 (MH2 + Pb) and Cd adsorbed on MH2 (MH2 + Cd) were analyzed by micro XRF, XRD, SEM/EDS, FTIR and XPS.

### 2.4. Immobilization of Pb/Cd in Soil

Smelting site soil samples in Baiyin, Gansu Province (36°33′ N, 104°13′ E) were collected. The air-dried soil samples were ground till they passed through a 2-mm sieve, then 0.1 g soil samples were digested by 17 M HNO_3_/8.8 M H_2_O_2_ [36] and the extracts were used to determine the metals content by ICP-OES. Basic properties of the soil are given in Table S1. The concentrations of Pb(II) and Cd(II) all exceeded the risk intervention values for soil contamination in GB 36600-2018 of China.

Air-dried soil samples (200 g each) were placed in 250 mL high density polyethylene (HDPE) cups at 25 °C with 5.0% MH2 addition, or 5.0% CMB addition, or without immobilizers (BLK). The soils in cups were wetted with deionized water to keep 70% of the field moisture capacity and then covered with tissue sealing film. The samples in each cup were collected after 1, 3, 15 and 25 days, dried at 30 °C and ground for further analysis. Soil pH and electrical conductivity (EC) were determined.

Bioavailable Pb(II) and Cd(II) concentrations of soil samples were extracted by CaCl_2_ solution: 1 g soil was mixed with 10 mL 0.1 M CaCl_2_ solution, and then the mixture was shaken at 240 ± 10 rpm for 2 h (25 °C). After CaCl_2_ extraction, the suspension was centrifuged (20 min, 11,000 rpm), filtered through 0.22-μm filters and analyzed by ICP-OES. The soluble Pb(II) and Cd(II) was extracted by deionized water likewise [37]. Every treatment was conducted with three parallel tests.

## 3. Results and Discussion

### 3.1. Material Characterization

#### 3.1.1. Basic Physicochemical Properties of Synthetic Materials

**Figure 1:**
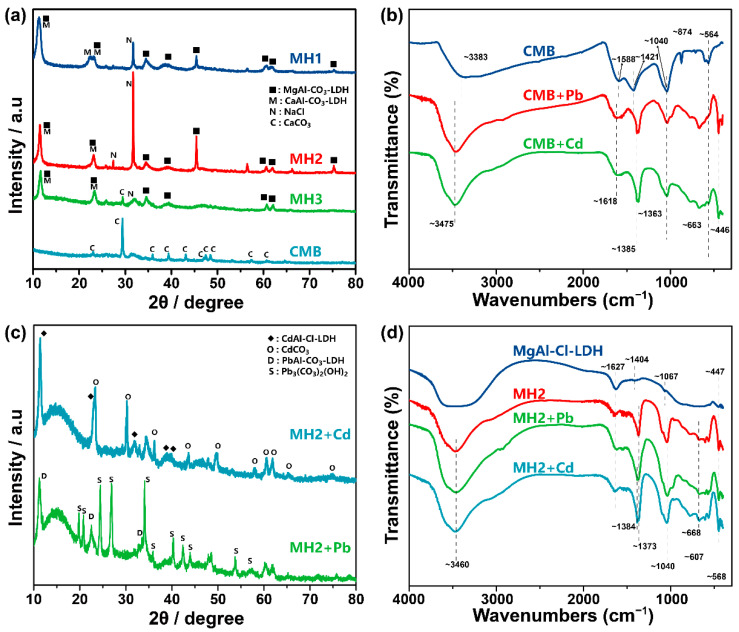
(**a**) XRD patterns of CMB, MH1, MH2 and MH3. (**b**) FTIR spectra of CMB before and after adsorption of Pb and Cd. (**c**) XRD patterns of MH2 after adsorption of Pb and Cd. (**d**) FTIR spectra of MgAl-Cl-LDH and MH2 before and after adsorption of Pb and Cd. XRD: X-ray diffraction. FTIR: Fourier Transform Infrared Spectroscopy. CMB: chicken-manure biochar. MH1, MH2, MH3 are the obtained MgAl-Cl-LDH-doped CMB prepared with 5 g, 15 g or 30 g CMB and 0.15 mol MgCl_2_ and 0.075 mol AlCl_3_.

**Figure 2:**
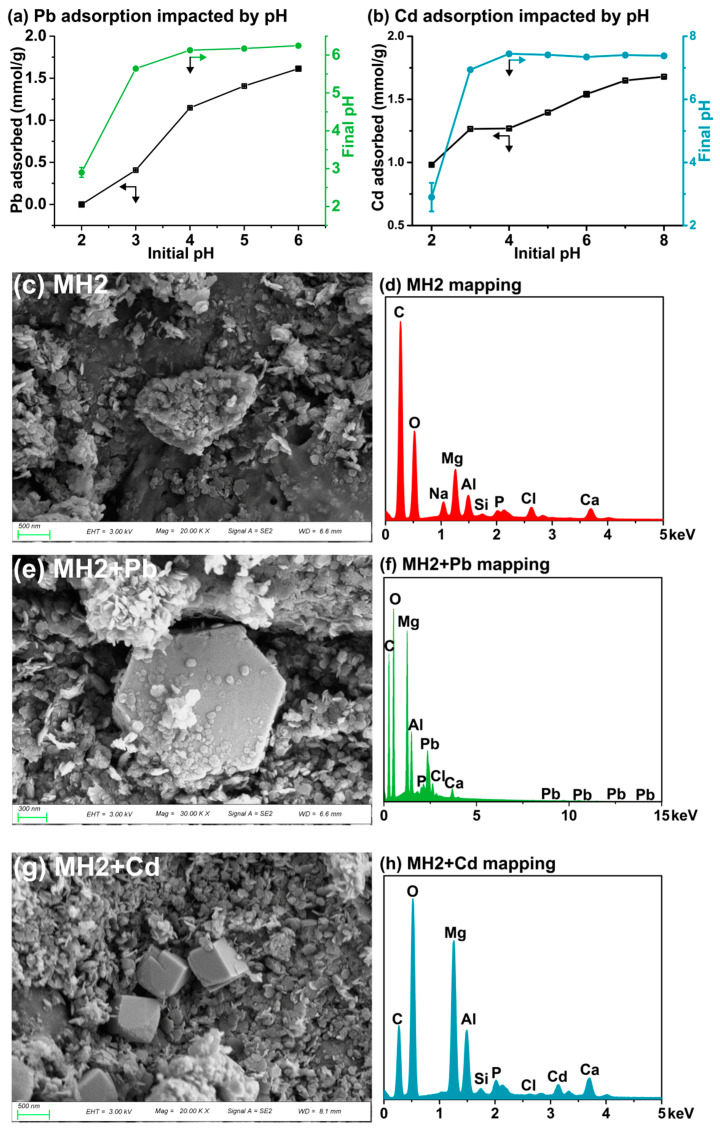
The effect of pH on adsorption of Pb (**a**) and Cd (**b**) onto MH2. SEM (**c**) and EDS mapping (**d**) of MH2, MH2 + Pb (**e**,**f**) and MH2 + Cd (**g**,**h**).

**Figure 3:**
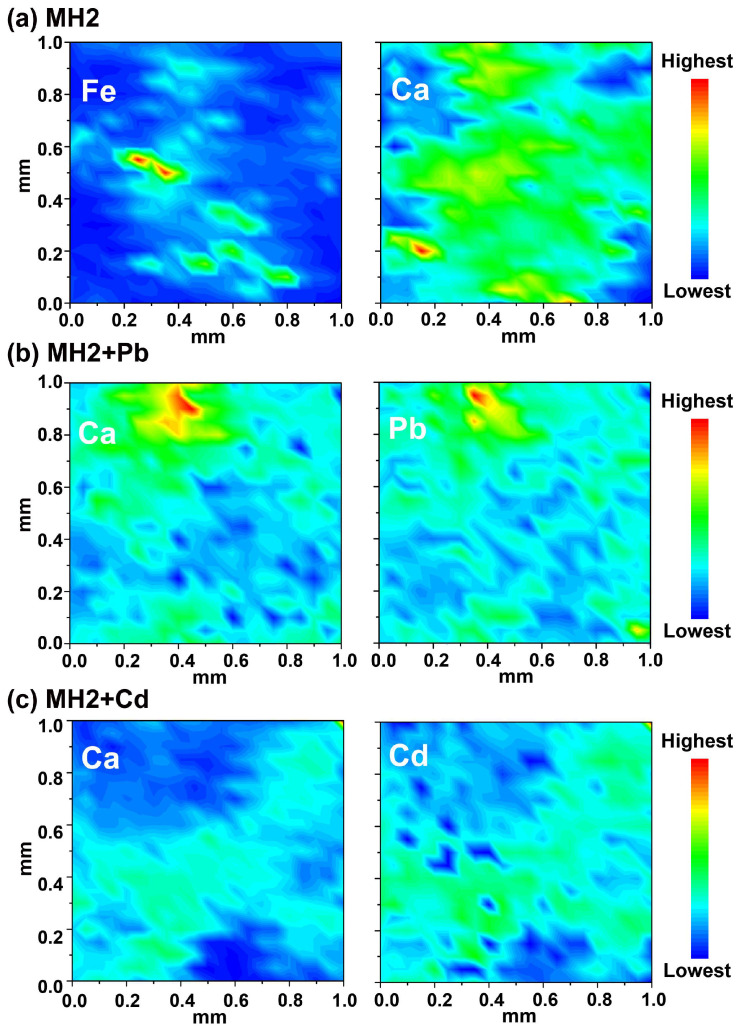
XRF graphs of MH2 (**a**), MH2 + Pb (**b**) and MH2 + Cd (**c**). Fe represents CMB content in MH2, C represents both CMB and LDH part in MH2.

XRD patterns of prepared CMB, MH1, MH2, and MH3 are presented in Figure 1a. CaCO_3_ (PDF#83-0577) was found in CMB [38]. After being doped with LDH, CaCO_3_ diffraction peaks could only be found in MH3 with the highest CMB addition. MH1, MH2 and MH3 all showed diffraction peaks of MgAl-CO_3_-LDH, suggesting the successful deposition of LDHs on CMB matrices. The diffraction peaks of CaAl-CO_3_-LDH were also observed (Figure 1a); these were formed by the Al(III) and Ca(II) originally contained in CMB [17]. The CO_3_^2−^ in LDH interlayers stemmed from CMB [26,39]. The absence of MgAl-Cl-LDH resulted from the much higher selectivity towards divalent anions than monovalent anions on LDH [39,40]. As far as we are concerned, this is the first study to combine two types of LDHs within one biochar matrix, providing more possibilities for Pb/Cd adsorption routes. Although P-containing minerals were not detected in XRD tests, EDS mapping (Figure 2d) and element analysis results (Table S2) of MH2 showed that traces of P contained in MH2. Likewise, traces of Fe were also found in XRF examination (Figure 3a). Significant correlations existed between Fe (represented in the CMB part) and Ca (represented in both CMB and LDH parts) according to Spearman matrix (ρ = 0.614) at the 0.01 level, indicating uniform distribution of LDH in CMB matrix.

Table S2 lists some physicochemical properties of the four materials. BET microSSA and micropore volume results (Table S2) proved that CMB contained large amounts of micropores instead of mesopores [41], which meant that CMB could offer more interfaces for the capture of contaminants than expected. The high C/N ratio and low H/C ratio in CMB were indicative of a high degree of carbonization and aromatization of the matrix [41]. Moreover, the weight percent of CMB (CMB%) in MH1, MH2 and MH3 was calculated to be 18.8%, 34.0% and 57.6%, which conformed to the incremental CMB dose during synthesis. According to the fitting result (Table S3) by the least squares method described in Text S2, the proposed linear model had a strong determinative ability for predicting CMB% based on designed CMB addition with satisfactory precision (R^2^ =1.00, root mean square error (RMSE) = 0.00165). Previous studies utilized complicated but ambiguous parameters to denote CMB%, only to reach an R^2^ less than 0.985 [31]. By contrast, this work offers a more accurate parameter to quantify the content of biochar in LDH dosed composites and assist future researchers to time-savingly predict the content of biochar before material characterization. As shown in the SEM graph (Figure 2c) of MH2, small LDH platelets were distributed in rough pores of CMB [42,43,44], which may be responsible for some loss of microSSA and micropore volume compared with CMB (Table S2).

The FTIR spectra of CMB (Figure 1b) were examined in order to identify the surface functional groups. Broad peaks around 3383 cm^−1^ were ascribed to the stretching vibration of hydroxyl groups [45]; apparent peaks at 1588, 1421 and 874 cm^−1^ represented aromatic C=C, aromatic C=C and aliphatic C-H, respectively, whereas the strong peak at 1040 cm^−1^ was assigned to the vibration of CO_3_^2−^ and aliphatic C-O [46], and 564 cm^−1^ was caused by O-P-O bond of PO_4_^3−^ [29]. MgAl-Cl-LDH was also put into FTIR analysis as a reference material. In addition to the peak at 1040 cm^−1^ shared by CMB, MH2 (Figure 1d) was found with more types of O-containing functional groups of LDH. The peak above 3000 cm^−1^ in MH2 became much broader due to LDH deposition [47], the peak at 1627 cm^−1^ was related to the water molecule in LDH [48], and the strong peak located at 1373 cm^−1^ was attributed to the stretching vibrations of CO_3_^2−^ in the interlayer of LDH and the remnant carbonate from CMB [47], and the indented adsorption peaks from 400 to 800 could be interpreted as the lattice vibration of M-O(H) in LDH layer [46,49,50].

TG analysis under N_2_ atmosphere further visualized organic and inorganic composition of CMB (Figure S2d) and MH2 (Figure S2e). For CMB, evaporation of adsorbed water and devolatilization of ash happened below 220 °C [51]. It was found that a slight mass loss appeared at 220–400 °C, in agreement with a small proportion of hemicellulose and cellulose in manure [52]. Temperatures of 400–600 °C witnessed a ~20% weight loss due to the decomposition of lignin [51]. A sharp weight loss above 600 °C was attributed to decomposition of CaCO_3_ [53]. In terms of MH2, the loss of adsorbed water and interlayer water occurred when temperature was below 210 °C. Dehydroxylation of M-OH was observed in 210–510 °C range along with the decomposition of hemicellulose and cellulose, where the mass loss in 320–400 °C was the prominent, corresponding to Mg-OH and Ca_2_Al-OH [54].

#### 3.1.2. Stability

Stability in water of CMB and MH2 was evaluated based on data illustrated in Figure S2a–c. The particle hydrodynamic diameters of fresh CMB and MH2 at pH 7 were 400–1300 and 450–1100 nm, respectively, but those values narrowed to 220–400 and 160–620 nm after 2 d due to electrostatic repulsion [55] (Figure S2c). The reduction rate of hydrodynamic size distribution and D_50_ for CMB and MH2 were recorded as 45–69%, 55%, 44–64% and 58% (Figure S2c), respectively, without apparent differences. However, the absolute value of zeta potential of CMB particles was constantly bigger than that of MH2 when pH > 6 (Figure S2a,b), implying that CMB would be more electrokinetically stable than MH2. On the contrary, at pH < 5, where the pH falls in real-life acid wastewater and smelting site soil [37,56], the absolute value of zeta potential of MH2 particles was bigger than that of CMB, indicating MH2 would be more electrokinetically stable than CMB.

Long-term stability in soils was measured by TG analysis in O_2_ (Figure S2f). R_50, MH2_ (95.5%) was nearly twice as much as R_50, CMB_ (54.1%), indicating that MH2 had an antioxidant property and the least biodegradation sensitivity [34]. The stability significantly improved by LDH doping indicated that stable organometallic complexes might be formed between MH2 and soil minerals to prevent the aging of biochar while immobilizing heavy metals [35].

### 3.2. Aqueous Adsorption of Pb/Cd

#### 3.2.1. Efficient Adsorption of Pb(II) and Cd(II) by LDH Doped CMB

The adsorption isotherm was fitted by Langmuir and Freundlich models, and the results are shown in Figure S3 and Table S4. According to R^2^ values, both Langmuir and Freundlich models fit the adsorption isotherm of Pb/Cd on CMB, MH1, MH2 and MH3, implying that different processes controlled the adsorption. The maximum adsorption capacity of CMB, MH1, MH2 and MH3 for Pb(II), estimated by Langmuir model, was 2.17, 1.95, 1.59 and 1.52 mmol/g, respectively, and the maximum adsorption capacity for Cd(II) was 0.497, 0.330, 0.650 and 0.642 mmol/g, respectively.

The adsorption capacity of MH2 to heavy metals did not increase linearly with the doping amount of LDH. Specifically, the relationship between relative Pb(II) (R_Pb_) and Cd(II) (R_Cd_) adsorption capacity and CMB% could be well fitted by polynomial response (Equations (5) and (6)) (R^2^ = 1.00) (Table S3). It was speculated that the following factors affect the adsorption of Pb(II) and Cd(II) by MH2: (1) the surface positive charges of LDH layers balanced a number of negative charges on CMB particles (Figure S2a,b) [57,58], which hampered electrostatic adsorption; (2) LDH particles occupied some adsorption sites that would otherwise accommodate Pb(II) and Cd(II). Although the Pb(II) adsorption capacities of MH1, MH2 and MH3 were lower than CMB, the adsorption capacities of MHs synthesized in this study exceeded that of most of the previously reported manure-based biochar [41,59].

R_Pb_ = 2.55 × CMB%^2^ − 2.48 × CMB% + 1.31 (5)

R_Cd_ = −11.1 × CMB%^2^ + 10.1 × CMB% − 0.841 (6)

Moreover, the variation trend of Cd(II) adsorption capacity of MH1, MH2 and MH3 was in line with that of microSSA and micropore volume, which suggested that physical adsorption caused by micropores might play a role in Cd(II) adsorption. Considering that Cd(II) has higher mobility and toxicity than Pb(II) in actual soils [60], the immobilization of Cd(II) is preferred. MH2 with maximum Cd(II) adsorption capacity was therefore selected for mechanism studies and immobilization application in soil studies.

The Pb/Cd adsorption capacity of MH2 was further compared with other modified manure-based biochar based on literature data [59,60,61,62,63,64,65,66] as listed in Table S7. MH2 exhibited excellent Pb adsorption capacities, which were 1.23 to 17.67 times higher than other types of manure-based biochar. And the Cd(II) adsorption capacity on MH2 was 1.35 to 65.00 times higher than that on previously modified manure-based biochar. For isothermal experiments for a binary Pb/Cd system, the results are shown in Figure S3i,j, similar to previous research [67,68], indicating that the existence of LDH in MH2 is helpful to immobilize Cd in the presence of Pb.

#### 3.2.2. Influence of Initial pH

The impact of initial pH (2.0–6.0) on Pb(II) adsorption onto MH2 is shown in Figure 2a. The final equilibrium pH climbed sharply when initial pH was at 2.0–4.0 range, and the final equilibrium pH was stable at around 6.2 (<pH_zpc_ (Figure S2a)) when pH was at 4.0–6.0 range. This meant that the surface of MH2 was protonated at the initial pH 2.0–4.0, which might obstruct electrostatic adsorption of Pb(II). However, the adsorption capacity increased sharply throughout the pH range (Figure 2a), revealing that electrostatic interaction was not the key factor to control adsorption of Pb(II). All the final pH values were above the diagonal line (Figure S4), indicating alkalization of the solution during adsorption, which was conducive to the immobilization of Pb(II) under acidic conditions [69].

Similarly, the effect of pH (2.0–8.0) on Cd adsorption onto MH2 was studied (Figure 2b). When initial pH was >3.0, the final pH (~7.4) value at equilibrium was higher than pH_zpc_ (~6.41) (Figure S2a), which favored electrostatic interaction. The final pH values were above the diagonal line (initial pH 2.0–7.0) (Figure S4), and the alkalization was conducive to the immobilization of Cd(II) [69].

#### 3.2.3. Pb/Cd Adsorption Mechanism of CMB

First, the mechanism of adsorption of Pb/Cd by CMB was studied. FTIR analysis (Figure 1b) were performed to characterize the changes of functional groups before and after Pb/Cd adsorption on CMB. Pb adsorbed on CMB (CMB + Pb) and Cd adsorbed on CMB (CMB + Cd) showed similar bands at close range. Typically, the broad peak at 3383 cm^−1^ in CMB was obviously narrowed and strengthened in CMB + Pb and CMB + Cd, and the peak shifted to 3475 cm^−1^, indicating that -OH complexed Pb/Cd [17]. Peaks at 1588 and 1421 cm^−1^ shifted to 1618 and 1385 cm^−1^ after adsorption, probably because Pb/Cd formed soft-soft acid-base bonds accompanied by aromatic ring C=C (cation-π bonding) [70]. The disappearance of peak at 874 cm^−1^ hinted that -CH might involve in the adsorption as well. The appearance of bands at 1363 cm^−1^ and weakening of bands at 1040 cm^−1^ also suggested the formation of metal-carbonates precipitates. Specifically, the band at 564 cm^−1^ in CMB shifted in CMB + Pb, representing the formation of lead phosphate.

The ion concentrations after adsorption equilibrium of Pb/Cd onto CMB is shown in Table S5. The adsorption amount of Pb(II) by CMB was significantly correlated with the release of Ca(II) (r = 0.989, *p* < 0.05), but the adsorption amount of Cd(II) was not correlated with Ca(II) (Figure S5a,c), indicating that cation exchange existed solely between Ca(II) and Pb(II). The pH_zpc_ of CMB was 4.78 (Figure S2a), lower than initial pH and final pH in adsorption experiments, which accounted for the electrostatic adsorption of Pb/Cd with negatively charged CMB. In summary, CMB adsorbed Pb(II) through complexation, cation exchange, precipitation and electrostatic adsorption, while adsorbed Cd complexation, precipitation and electrostatic adsorption.

#### 3.2.4. Isomorphous Substitution and Precipitation Facilitate Long-Term Immobilization of Pb/Cd by MHs

The XRD pattern of MH2 + Pb is shown in Figure 1c. After the adsorption of Pb(II), a series of well-developed basal (00l) diffraction peaks was observed, indicating the formation of layer-by-layer crystal structures of PbAl-CO_3_-LDH, consistent with previous research on the isomorphic substitution of Pb(II) towards Mg(II) in LDH-doped biochar [71]. The significant correlation (r = 0.992, *p* < 0.05) between release of Mg(II) and adsorption amount of Pb(II) in solution at equilibrium (Table S5 and Figure S5b) supported the isomorphic substitution. In addition, SEM/EDS (Figure 2e,f) also observed a large plate, which might be the Pb-containing LDH. Importantly, the existence of Pb_3_(OH)_2_CO_3_ was confirmed by XRD (Figure 1c), and the precipitation of Pb(II) by MH2 was attributed to interlayer CO_3_^2−^ in LDH interlayer and the residual CaCO_3_ from CMB. Pb(II) in the form of PbAl-CO_3_-LDH is very stable because Pb(II) exists in the lattice of isomorphous substitutes. Pb_3_(OH)_2_CO_3_, as a well crystallized precipitate, is also stable in soil. Therefore, MH2 has the potential to immobilize Pb/Cd in soil for a long time. XRF mapping also showed a highly pairwise correlation between Ca(II) and Pb(II) in MH2 + Pb (Figure 3b) (Spearman correlation test at 0.01 level (ρ = 0.411)), indicating the calcium-containing substance in MH2 was the main phase for Pb(II) immobilization.

**Figure 4:**
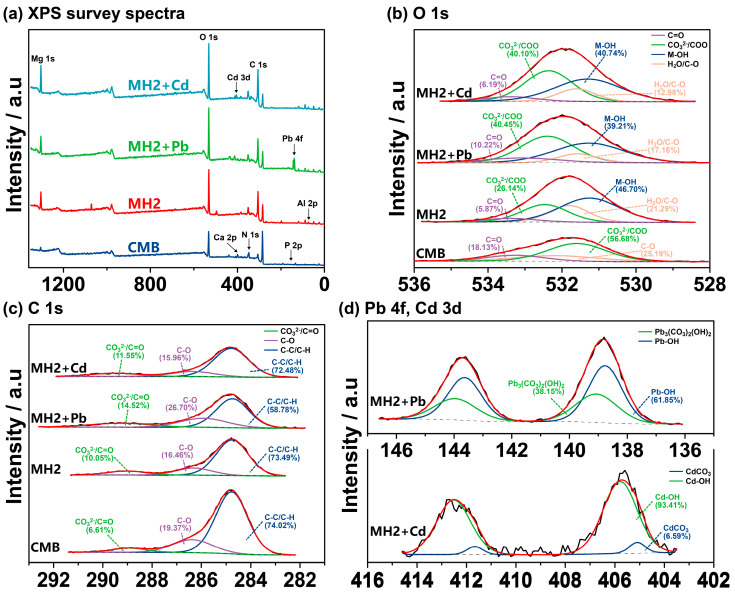
XPS survey spectra (**a**), high-resolution O 1s spectra (**b**) and C1s XPS spectra (**c**) of CMB, MH2, MH2 + Pb and MH2 + Cd. Pb 4f and Cd 3d spectra of MH2 + Pb and MH2 + Cd (**d**).

The total spectra of XPS are shown in Figure 4a, with a typical Pb 4f peak appearing in MH2 + Pb. Based on Figure 4b, the Pb(II) adsorption mainly involved carbonate/COO and C=O, which precipitated and complexed Pb(II) [17]. The decrease in content of H_2_O/C-O was associated with the varied water-absorbing capacity of PbAl-CO_3_-LDH and MgAl-CO_3_-LDH (Table S6), which indirectly proved the isomorphic substitution. The electron binding energy of Pb 4f in MH2 + Pb also pointed to the formation of Pb-containing LDH, Sur-OH-Pb^2+^ and Pb_3_(OH)_2_CO_3_ (Figure 4d) [72,73,74]. FTIR spectra of MH2 and MH2 + Pb showed merely slight differences, namely the appearance of peak at 1384 cm^−1^ after adsorption, corresponding to cation-π bonding by CMB part in MH2.

For Cd(II) adsorption on MH2, micro XRF mapping showed a uniform distribution of Cd(II) and Ca(II) on the surface (Spearman correlation test, ρ = 0.742 significant at 0.01 level) (Figure 3c), indicating that the Ca-containing phase played a non-negligible role. Specifically, as shown in Figure 1c and Figure 4d, CdCO_3_ and CdAl-Cl-LDH were the main mineral precipitates in MH2 + Cd. The formed CdCO_3_ on the surface of MH2 could also be observed by SEM (Figure 2g) [75], probably due to the high concentration of CO_3_^2−^ in MH2. The Gibbs free energy change of isomorphous substitution between Cd(II) and Ca(II) reflected the spontaneity of the reaction (∆G= −0.34 eV) [28]. The significant correlation (r = 0.951, *p* < 0.05) between the release of Ca(II) and adsorption amount of Cd(II) at equilibrium also reflected isomorphic substitution between Cd(II) and Ca(II) in the LDH layer (Table S5 and Figure S5). Cd(II) in the form of CdAl-Cl-LDH and CdCO_3_ would be very stable in the soil because Cd (II) exists in the lattice of isomorphous substitutes and crystalline precipitates.

The bands at 3460, 1384, 1373 and 1040 cm^−1^ in FTIR results (Figure 1d) after adsorption were attributed to the Cd(II) complexation by hydroxyl groups, cation-π bonding, precipitation and isomorphic substitution involved with carbonate.

### 3.3. Immobilization of Pb/Cd in Soil

#### 3.3.1. The Improvement on Physicochemical Properties of Soil

**Figure 5:**
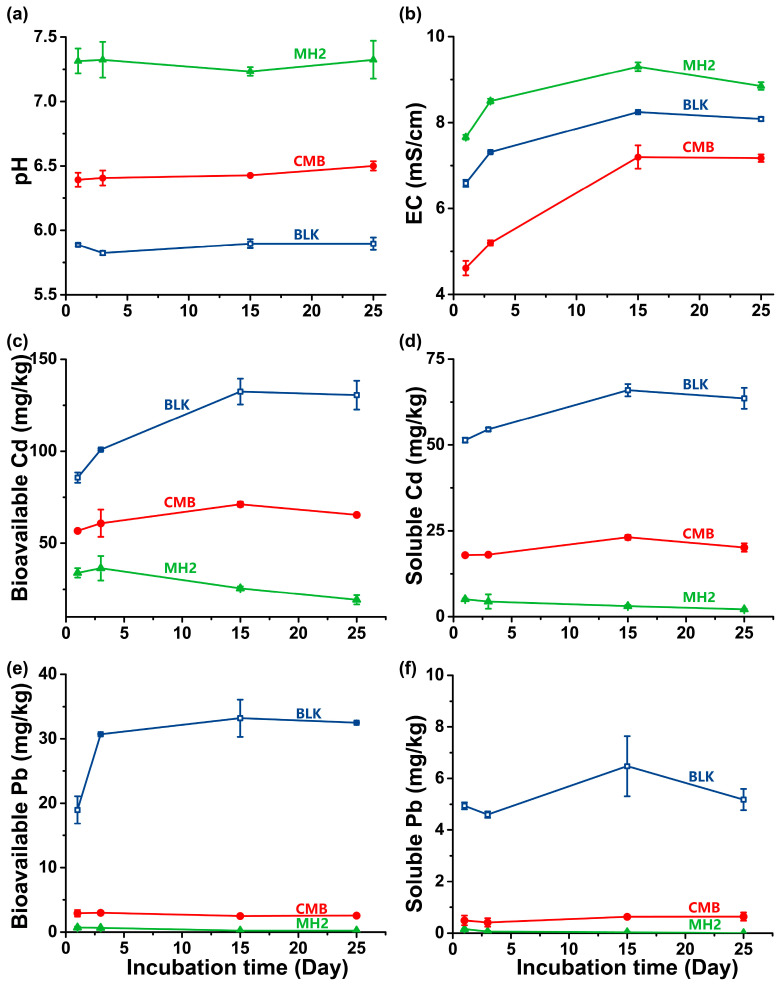
The pH (**a**) and EC (**b**) variations in the soil amended by CMB or MH2. Bioavailable concentrations of Cd (**c**), Pb (**e**) and water-extractable Cd (**d**), Pb (**f**) in the soil during CMB or MH2 amendment.

The pH and EC values, determined during the incubation experiment with the application of 5% CMB and 5% MH2, are presented in Figure 5a,b. MH2 was more alkaline than CMB (Table S2). After 25-d-application of the MH2, the soil pH increased from 5.9 to 7.7, and the EC also improved slightly (from 8.1 to 8.9 mS/cm), which would be conducive to the precipitation of mobile Cd(II) in soil [3]. On the contrary, CMB showed limited potential for amending soil acidity (increasing soil pH by only 0.6 in 25 d). The EC value in CMB-treated soil (4.6 to 7.2 mS/cm) was constantly lower than that of the control (6.6 to 8.1 mS/cm), which may be caused by the aging of biochar. Li et al. [76] found that wheat-straw biochar aged in vegetable fields could significantly reduce the soil EC by 4–15% within 44 d.

#### 3.3.2. Immobilization Efficiency in Soil

The application of MH2 greatly reduced the content of bioavailable Pb/Cd and water-soluble Pb/Cd in soil (Figure 5c,e). To be specific, compared with the BLK, the application of CMB reduced the content of bioavailable Cd in soil by 49.8%, while MH2 decreased the content of bioavailable Cd by 85.2%. Similarly, compared with the blank, the bioavailable Pb content in CMB and MH2-treated soil declined by 86.5% and 98.8%, respectively (Figure 5c,e). Similar results were observed in water-extractable Pb/Cd (Figure 5d,f). Compared with the blank, the soluble Pb concentrations in CMB and MH2-treated soil decreased by 87.7% and 99.5%, and soluble Cd concentrations decreased by 68.4% and 96.7%, respectively. Therefore, in 25 d, MH2 can achieve more efficient simultaneous immobilization of Pb/Cd in the smelting site soil than CMB. Further Pb/Cd immobilization by MH2 under field conditions may be lower than that observed in this test, but immobilizers may be applied for several times in longer time field experiments. Hence the results in this paper still provide valuable refences for the application of MH2. In addition, the stability of MH2 in fields should be monitored in the future.

## 4. Conclusions

In this study, a series of MHs were successfully synthesized. In a long-term stability test, R_50_ of MH2 was nearly twice as much as that of CMB, indicating that MH2 had antioxidant property and the least biodegradation sensitivity. In aqueous batch adsorption experiments, LDH-doped CMB slightly affected Pb(II) adsorption because surface positive charges of LDH layers balanced part of negative charges on CMB, and LDH particles occupied some physical adsorption sites. The doping of LDH effectively improved the adsorption performance of MHs on Cd(II). The adsorption capacity of Cd(II) on MH2 was 3.14 times higher than that on CMB. The isomorphic substitution and precipitation were the key mechanisms of MH2 immobilizing Pb/Cd, which formed stable Pb/Cd species, i.e., PbAl-CO_3_-LDH, Pb_3_(OH)_2_CO_3_, CdAl-Cl-LDH, and CdCO_3_. The application of 5% MH2 markedly reduced the content of bioavailable Pb/Cd and water-soluble Pb/Cd in soil. After 25 d incubation, the contents of bioavailable Pb and Cd decreased by 98.8% and 85.2%, respectively, and the contents of soluble Pb and Cd declined by 99.5% and 96.7%. This study provides new insights for the synthesis of functional biochar containing materials with long-term stability of immobilized Pb/Cd.

## Supplementary Materials

The following supporting information can be downloaded at: https://www.mdpi.com/article/10.3390/ijerph20010867/s1. Figure S1: Flow chart of layered double hydroxide doped chicken-manure biochar (CMB) synthesis. Figure S2: Zeta potential, particle size distribution and thermogravimetric results of CMB and MH2. Figure S3: The Pb/Cd adsorption isotherm of CMB ((a) and (b)), MH1 ((c) and (d)), MH2 ((e) and (f)), and MH3 ((g) and (h)) fitted by Langmuir and Freundlich models. Simultaneous adsorption of (i) Pb and (j) Cd by MH2 in binary metal systems. Figure S4: The relationship of final pH and initial pH in adsorption of Pb/Cd onto MH2. Figure S5: The correlations between ion concentrations at adsorption equilibrium of Pb/Cd onto CMB and MH2. * *p* < 0.01 (two-tailed). Table S1: Basic properties of Baiyin smelting site soil. Table S2: Basic Physicochemical Properties of CMB, MH1, MH2 and MH3. Table S3: Fitting parameters for the composition of products and Pb/Cd optimal design. Table S4: Fitting parameters for Pb(II) and Cd(II) adsorption isotherm. Table S5: Ion concentrations in aqueous solutions after adsorption equilibrium of Pb/Cd onto CMB and MH2. Table S6: Binding energies and atom ratios of C 1s, O 1s, Pb 4f and Cd 3d peaks on CMB, MH2, MH2 with Pb and MH2 with Cd. Table S7: Summary of modified manure-based biochar adsorption capacities for Pb(II) and Cd(II). Text S1: Preparation of layered double hydroxide doped chicken-manure biochar. Text S2: The relationship of the product composition and adsorption capacities of Pb/Cd. Text S3: The evaluation of Pb/Cd adsorption capacity of MH2. References [31,33,59,60,61,62,63,64,65,66,67,68] are cited in the supplementary materials.
