# Supplementary material for: Potential Mechanism of Long-Term Immobilization of Pb/Cd by Layered Double Hydroxide Doped Chicken-Manure Biochar
Source: Int J Environ Res Public Health. 2023 Jan 3;20(1):867. doi: 10.3390/ijerph20010867 (PMC9819711; doi:10.3390/ijerph20010867)
Supplement: Supplementary file 1 [file ijerph-20-00867-s001.zip › ijerph-2105465-supplementary.pdf]

Supplementary Materials

Potential Mechanism of Long-Term Immobilization of Pb/Cd by Layered Double Hydroxide Doped Chicken-Manure Biochar

Xiaoxian Zhang [1], Tingran Liu [1], Jichen Zhang [1] and Ling Zhu [1,*]

[1] Department of Environmental Science, College of Environmental Science and Engineering, State Key Laboratory of Pollution Control and Resources Reuse, Shanghai Institute of Pollution Control and Ecological Security, Tongji University, Shanghai 200092, China; clarechang@tongji.edu.cn (X.Z.); 2130518@tongji.edu.cn (T.L.); 2030532@tongji.edu.cn (J.Z.); zhu_ling@tongji.edu.cn (L.Z.).

* Correspondence: zhu_ling@tongji.edu.cn

Number of pages: 17.

Number of tables: 7.

Number of figures: 5.

Text S1. Preparation of layered double hydroxide doped chicken-manure biochar……………………………………S3

Figure S1. Flow chart of layered double hydroxide doped chicken-manure biochar synthesis..…………………………S4

Text S2. The relationship of the product composition and adsorption capacities of Pb/Cd………………………………S5

Table S1. Basic properties of Baiyin smelting site soil..………………..………………………………………………S6

Table S2. Basic Physicochemical Properties of CMB, MH1, MH2 and MH3…………………………………………S7

Table S3. Fitting parameters for the composition of products and Pb/Cd optimal design...…………………………..….S8

Table S4. Fitting parameters for Pb(II) and Cd(II) adsorption isotherm.....…………………………………………S9

Figure S2. Zeta potential, particle size distribution and thermogravimetric results of CMB and MH2..………………S10

Figure S3. The Pb/Cd adsorption isotherm of CMB ((a) and (b)), MH1 ((c) and (d)), MH2 ((e) and (f)), and MH3 ((g) and (h)) fitted by Langmuir and Freundlich models. Simultaneous adsorption of (i) Pb and (j) Cd by MH2 in binary metal systems……………………………………………………………………………………….………………S11

Figure S4. The relationship of final pH and initial pH in adsorption of Pb/Cd onto MH2………………………………S12

Table S5. Ion concentrations in aqueous solutions after adsorption equilibrium of Pb/Cd onto CMB and MH2….…S13

Figure S5. The correlations between ion concentrations at adsorption equilibrium of Pb/Cd onto CMB and MH2.…S14

Table S6. Binding energies and atom ratios of C 1s, O 1s, Pb 4f and Cd 3d peaks on CMB, MH2, MH2 with Pb and MH2 with Cd..………S15

Table S7. Summary of modified manure-based biochar adsorption capacities for Pb(II) and Cd(II)..…………………S16

Text S3. The evaluation of Pb/Cd adsorption capacity of MH2….……………………………………………………S17

Text S1. Preparation of layered double hydroxide doped chicken-manure biochar.

0.15 mol $MgCl_2$ and 0.075 mol $AlCl_3$ were resolved in a 1000 mL three-neck flask, and 5 g, 15 g or 30 g chicken-manure biochar (CMB) [31] were added under vigorous stirring for 45 min. The initial pH values for the 3 trials were 1.2. Then pH value was adjusted to approximately 9.5 by 2 mol/L NaOH dropwise, then the mixture was aged at 80°C for 18 h [33]. The above process was conducted with N_2 flow. After cooled to room temperature, the precipitates were washed with deionized water and centrifuged for 8 times, and the resulting composites were dried at 80°C for 12 h. The prepared materials were referred to MH1, MH2, and MH3.

Figure S1. Flow chart of layered double hydroxide doped chicken-manure biochar synthesis.

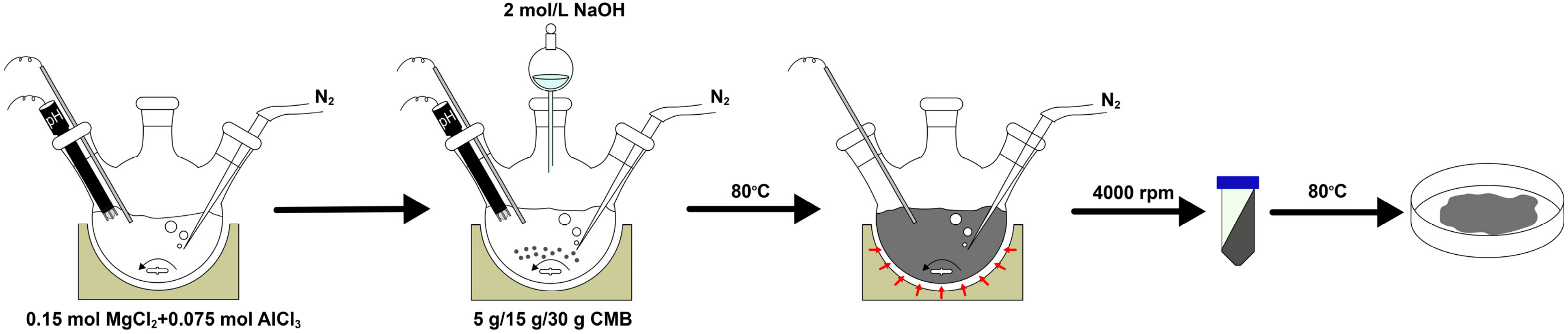

Text S2. The relationship of the product composition and adsorption capacities of Pb/Cd.

The least squares method for linear regression was used to fit the experimental data on the relationship of CMB% (described in **2.1.**) and CMB addition. The parameters a and b in Equation S4 were obtained easily, and root mean square error (RMSE) and coefficient of determination (R^2) were calculated to assess the method afterwards. And non-linear method for non-linear regression was used by solver add-in function of the Microsoft Excel (2016) on the relationship of relative Pb adsorption capacity (R_{Pb}) and CMB%, as well as the relationship of relative Cd adsorption capacity (R_{Cd}) and CMB%. R^2 of the fitting models was recorded.

$$CMB\% = a \cdot m + b, \quad (S1)$$

where CMB% is the mass ratio of CMB in the product, m is the addition of CMB (g) in the synthesis of LDH doped CMB, and a (%/g) and b (%) are fitting parameters.

Table S1. Basic properties of Baiyin smelting site soil.

pH (H_2O)	EC[1] (mS/m)	Total contents of metals (mg/kg)						
		**Pb**	**Cd**	**As**	**Ca**	**Mn**	**Al**	**Mg**
8.37	703.91	5866.0	166.2	192.6	66737.1	1071.9	20600.2	13013.2

[1] EC, electrical conductivity.

Table S2. Basic Physicochemical Properties of CMB, MH1, MH2 and MH3.

**Sample**	**CMB**	**MH1**	**MH2**	**MH3**
pH	8.98	8.91	9.32	9.65
MicroSSA* (m^2/g)	114.7	45.57	48.22	29.68
Micropore volume (cm^3/g)	0.068	0.034	0.039	0.030
C (%)	38.56	8.47	14.22	23.4
H (%)	2.56	3.36	2.67	3.00
N (%)	2.49	0.47	0.85	1.43
H/C	0.07	0.40	0.19	0.13
C/N	15.49	18.19	16.78	16.31
Ca (%)	10.55	2.31	4.94	7.40
Mg (%)	1.71	14.83	9.87	8.93
Al (%)	0.03	5.77	4.56	4.09
Fe (%)	0.06	/***	/***	0.01
Na (%)	0.16	1.99	7.35	0.40
P (%)	4.16	0.68	2.12	3.57
K (%)	0.56	0.11	0.16	0.14
CMB (%)**	100	18.8	34.0	57.6

* MicroSSA, specific surface area of micropore materials.

** CMB (%) was calculated according to *2.1 Material preparation and characterization*.

*** Below detection limit of ICP-OES.

Table S3. Fitting parameters for the composition of products and Pb/Cd optimal design.

Parameters		CMB	MH1	MH2	MH3
CMB%		100	18.8	34.0	57.6
d^2		/	1.96×10^{-6}	2.56×10^{-6}	3.61×10^{-6}
a		1.55×10^{-2}			
b		1.09×10^{-1}			
root mean square error (RMSE)		$1.65*10^{-3}$			
R^2		1.00			
Pb	R_{Pb} (%)	100	93.0	75.7	72.3
	R^2*	1.00			
Cd	R_{Cd} (%)	100	66.4	1.31	1.29
	R^2**	1.00			

* R^2, parameter on the relationship of relative Pb adsorption capacity (R_{Pb}) and CMB% based on **Text S2**.

** R^2, parameter on the relationship of relative Cd adsorption capacity (R_{Cd}) and CMB% based on **Text S2**.

Table S4. Fitting parameters for Pb(II) and Cd(II) adsorption isotherm.

Parameters		CMB	MH1	MH2	MH3
Pb	Q_m (mmol/g)	2.17	1.95	1.59	1.52
	K_L (L/g)	16.0	572	1240	169
	Langmuir R^2	0.934	0.991	0.921	0.953
	K_F ($g^{(1-n)}/L^{-n}/g$)	2.10	1.85	1.56	1.69
	n	170	12.3	29.4	7.36
	Freundlich R^2	0.989	0.911	0.925	0.930
Cd	Q_m (mmol/g)	0.497	0.330	0.650	0.642
	K_L (L/g)	28.7	130	79.7	292
	Langmuir R^2	0.969	0.989	0.819	0.991
	K_F ($g^{(1-n)}/L^{-n}/g$)	0.483	0.355	0.707	0.677
	n	8.82	9.89	9.01	15.8
	Freundlich R^2	0.967	0.997	0.987	0.959

Figure S2. Zeta potential, particle size distribution and thermogravimetric results of CMB and MH2.

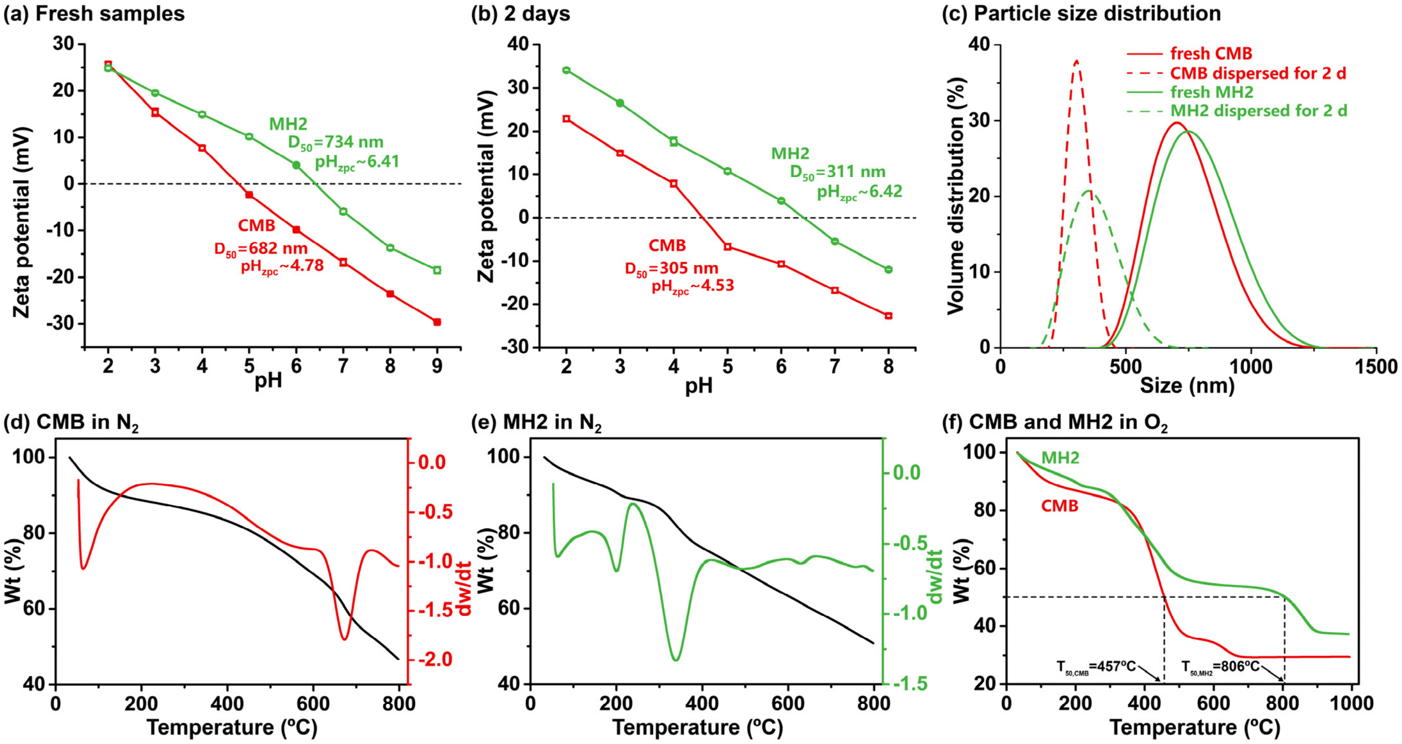

Figure S3. The Pb/Cd adsorption isotherm of CMB ((a) and (b)), MH1 ((c) and (d)), MH2 ((e) and (f)), and MH3 ((g) and (h)) fitted by Langmuir and Freundlich models. Simultaneous adsorption of (i) Pb and (j) Cd by MH2 in binary metal systems.

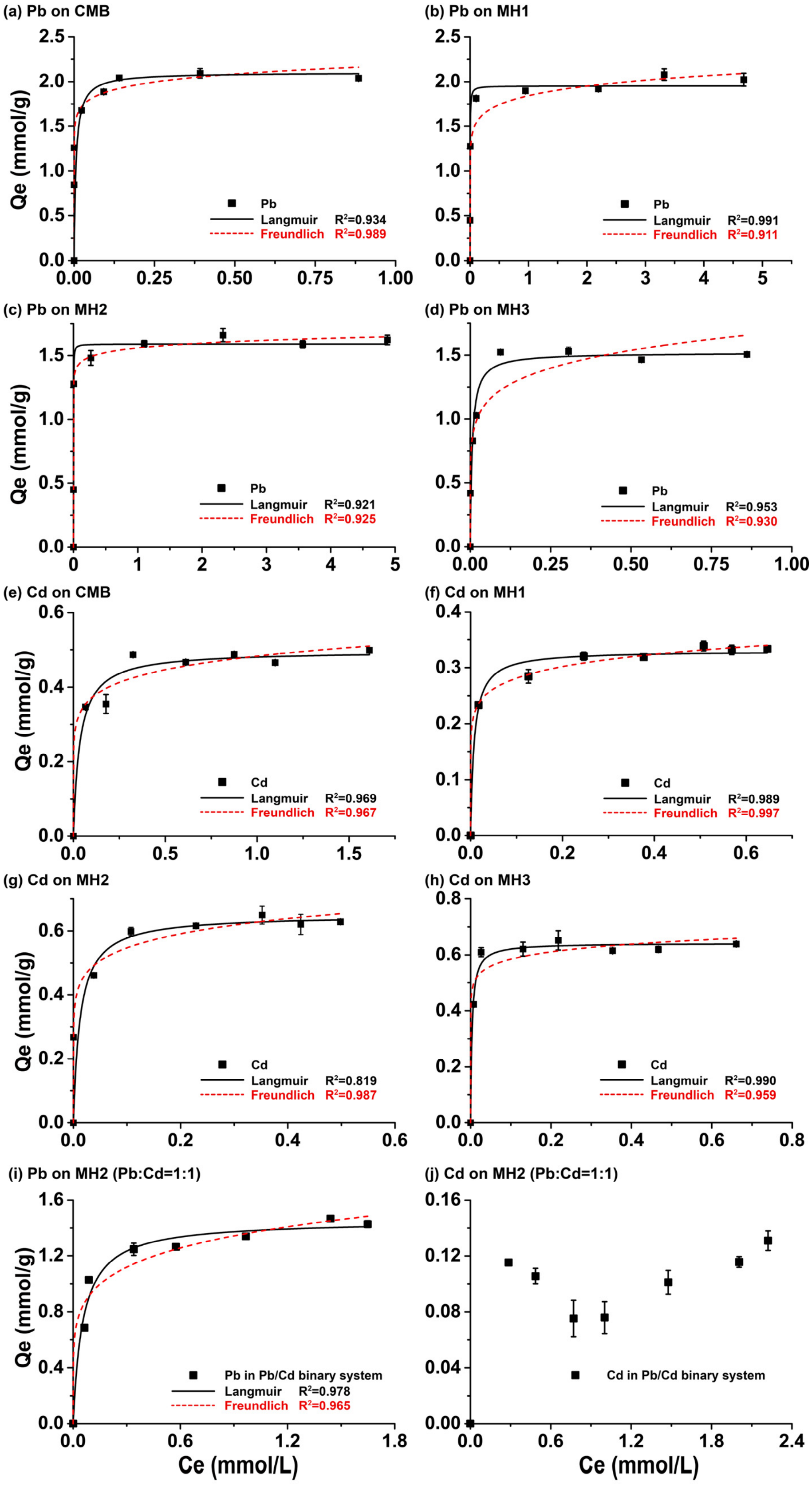

Figure S4. The relationship of final pH and initial pH in adsorption of Pb/Cd onto MH2.

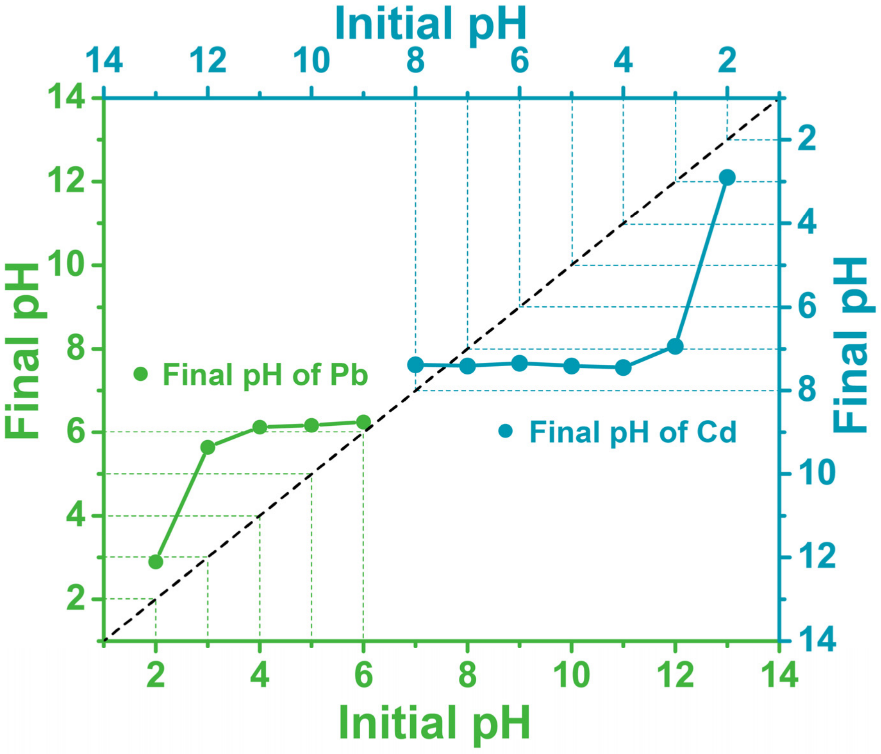

Table S5. Ion concentrations in aqueous solutions after adsorption equilibrium of Pb/Cd onto CMB and MH2.

Conc. * (mg/L) / adsorbent	Pb	Ca in Pb solutions	Mg in Pb solutions	Cd	Ca in Cd solutions	Mg in Cd solutions
CMB	0.00	14.9	4.4	7.4	5.9	3.5
	0.01	22.2	4.3	19.9	6.0	3.0
	4.84	26.9	3.7	36.4	5.7	3.6
	19.1	27.8	4.0	68.7	6.0	4.0
	29.1	29.8	4.0	98.4	5.4	3.8
	81.3	30.8	3.4	123.4	6.0	3.9
	266.2	30.5	4.1	181.1	5.8	3.7
MH2	0.00	8.4	0.5	0.0	3.0	0.9
	0.68	9.5	3.7	4.3	3.5	0.7
	56.0	9.4	4.9	12.1	4.3	0.7
	228.1	8.2	5.8	25.7	4.4	0.8
	481.8	8.3	5.8	39.6	4.5	0.8
	738.8	8.4	5.8	47.7	4.4	0.9
	1011.3	8.0	5.5	56.0	4.0	0.6

* Conc., Concentration.

Figure S5. The correlations between ion concentrations at adsorption equilibrium of Pb/Cd onto CMB and MH2. *P< 0.01 (two-tailed).

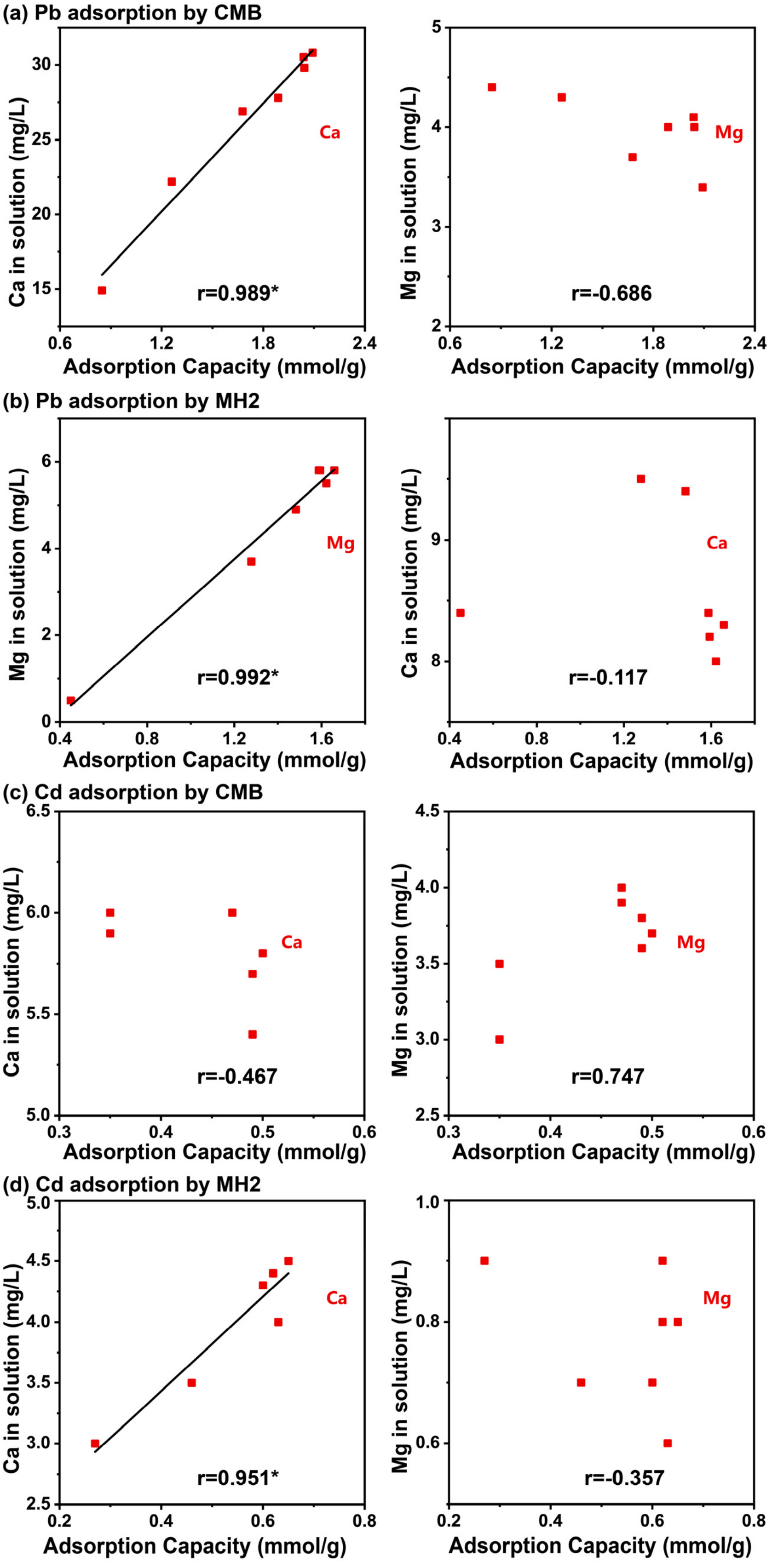

Table S6. Binding energies and atom ratios of C 1s, O 1s, Pb 4f and Cd 3d peaks on CMB, MH2, MH2 with Pb and MH2 with Cd.

Item	O 1s Peak	BE*/ eV	Percent/ %	C 1s Peak	BE*/ eV	Percent/ %	Pb 4f7/2 or Cd 3d5/2 Peak	BE/eV	Percent/%
CMB	C=O	533.3	18.1	CO_3^{2-}/C=O	288.9	6.6	/		
	CO_3^{2-}/COO	531.6	56.7	C-O	286.4	19.4			
	C-O	532.2	25.2	C-C	284.8	74.0			
MH2	C=O	533.4	5.9	CO_3^{2-}/C=O	289.1	10.1	/		
	CO_3^{2-}/COO	532.5	26.1	C-O	286.3	16.5			
	H_2O/C-O	531.8	21.3	C-C	284.8	73.5			
	M-OH	531.3	46.7	/					
MH2 +Pb	C=O	533.1	10.2	CO_3^{2-}/C=O	289.3	14.5	$Pb_3(CO_3)_2(OH)_2$	139.1	38.2
	CO_3^{2-}/COO	532.4	40.5	C-O	285.9	26.7	Pb-OH	138.8	61.9
	H_2O/C-O	531.6	10.1	C-C	284.8	58.8	/		
	M-OH	531.3	39.2	/					
MH2 +Cd	C=O	533.4	6.2	CO_3^{2-}/C=O	289.5	11.6	Cd-OH	405.8	93.4
	CO_3^{2-}/COO	532.4	40.1	C-O	286.3	16.0	CdCO3	405.1	6.6
	H_2O/C-O	531.6	13.0	C-C	284.8	72.5	/		
	M-OH	531.3	40.7	/					

* BE, Binding energy.

Table S7. Summary of modified manure-based biochar adsorption capacities for Pb(II) and Cd(II).

Material	Heavy metals	Solution pH	Maximum adsorption capacity (mmol/g)	References
MH2	Pb(II)	5.0	1.59	This study
H_2O_2 modified swine manure biochar	Pb(II)	7.0	0.09	[61]
HNO_3 modified swine manure biochar	Pb(II)	5.0	0.24	[62]
Fe^{2+}/Fe^{3+} modified swine manure biochar	Pb(II)	5.0	0.26	[62]
H_3PO_4 modified CMB	Pb(II)	5.0	0.27	[60]
CMB co-pyrolyzed with Chinese medicine	Pb(II)	6.5	0.77	[63]
NaOH modified dairy manure biochar	Pb(II)	6.0-7.0	0.93	[64]
MnO_2 modified swine manure biochar	Pb(II)	5.0	1.29	[59]
MH2	Cd(II)	5.0	0.65	This study
H_2O_2/3-mercaptopropyltrimethoxysilane modified swine manure biochar	Cd(II)	7.0	0.01	[61]
H_3PO_4 modified CMB	Cd(II)	5.0	0.07	[60]
Swine manure biochar	Cd(II)	6.0	0.19	[65]
Silkworm manure biochar	Cd(II)	6.0	0.19	[65]
NaOH modified dairy manure biochar	Cd(II)	6-7	0.36	[64]
MnO_2 modified swine manure biochar	Cd(II)	5.0	0.41	[59]
$KMnO_4$ modified vermicompost biochar	Cd(II)	5.5	0.48	[66]

Text S3. The evaluation of Pb/Cd adsorption capacity of MH2.

The Pb/Cd adsorption capacity of MH2 was compared with other modified manure-based biochar in accordance with the literature data (Table S7). MH2 exhibited excellent Pb adsorption capacities, which were 1.23 to 17.67 times higher than other manure-based biochar. And the Cd(II) adsorption capacity on MH2 was 1.35 to 65.00 times higher than that on previously modified manure-based biochar. Therefore, MH2 is promising in application to single-component Pb/Cd adsorption.

Considering the complicated heavy metal components in real-life environment, Pb(II) and Cd(II) were iso-stoichiometrically mixed with MH2. Figure S3 (i) and (j) show the results of binary isothermal experiments. The maximum adsorption capacity of MH2 for Pb(II) in binary Pb/Cd system estimated by Langmuir model reached 1.46 mmol/g, similar to that in single Pb system. However, the adsorption of Cd(II) by MH2 was strongly affected by the coexisting Pb(II). Cd(II) adsorption capacity fluctuated from 0.08 to 0.12 mmol/g when initial Pb concentration increased to 2.4 mmol/L. The trend of Cd(II) isotherm in binary Pb/Cd system was analogous to literature [60,67,68], suggesting that adsorption sites were probably occupied with Pb(II) species with more affinity and/or precipitation of biochar and LDH. The presence of Pb(II) may cost excessive carbonate so that the formation of $CdCO_3$ is inhibited, leaving isomorphic substitution the dominate mechanism in binary Cd(II) adsorption. Thanks to the formation of both MgAl-CO_3-LDH and CaAl-CO_3-LDH, the isomorphic substitution of Pb and that of Cd are individually taking place, allowing for a higher Cd immobilizing efficiency in the presence of Pb.
